# Combating Drug‐Resistant Tuberculosis: Advancements in Diagnostics and Therapeutics

**DOI:** 10.1155/pm/3346306

**Published:** 2026-08-17

**Authors:** Silvi Gautam, Jahanvi Saini, Shabaaz Begum Jameel Pasha, Divakar Sharma, Divya Venugopal

**Affiliations:** ^1^ Department of Microbiology, Graphic Era (Deemed to be) University, Dehradun, India, geu.ac.in; ^2^ Department of Biotechnology, Graphic Era (Deemed to be) University, Dehradun, India, geu.ac.in

**Keywords:** antibiotics, drug repurposing, drug resistance, mutation, *Mycobacterium tuberculosis*, nanotechnology

## Abstract

The increasing prevalence of multidrug‐resistant (MDR) and extensively drug‐resistant (XDR)*Mycobacterium tuberculosis* has emerged as a major global health concern. Advanced molecular diagnostics, including rapid assays, have improved resistance detection; however, resource‐limited settings remain underserved. Nanotechnology‐based drug delivery systems, including polymeric carriers, solid lipid nanoparticles, and silver nanoparticles, can provide targeted delivery, prolonged release, and improved bioavailability. Drug repurposing strategies using existing drugs like linezolid, celecoxib, and nitazoxanide also show a promising option in combating drug‐resistant TB while reducing treatment duration. Host‐directed therapies (HDTs) and natural products offer complementary strategies by enhancing host immune responses and providing multitarget antimycobacterial effects. This review emphasizes the drug resistance mechanisms and advancements in novel theranostics. Despite significant progress, challenges related to cost‐effective scalability, accessibility of advanced diagnostics, and optimization of therapeutic systems continue to limit clinical translation. Future research must prioritize multidisciplinary collaborations to accelerate drug development, enhance diagnostic capabilities, and expand nanotechnology‐based solutions to address the TB epidemic effectively.

## 1. Introduction

Tuberculosis (TB), caused by *Mycobacterium tuberculosis*, remains a major global health burden, particularly in low‐ and middle‐income countries [1]. An estimated 10.8 million people become ill from TB, and approximately 1.25 million people die from it, making it a leading cause of death among infectious diseases [1]. The condition particularly impacts people in 30 high‐burden countries, which include Indonesia (10%), India (26%), Pakistan (6.3%), China (6.8%), the Philippines (6.8%), and collectively account for 56% of global TB cases [1]. MTB enters the pulmonary alveoli, where macrophages engulf it and have developed adaptations for survival within macrophages, avoiding phagosome formation and resisting host defenses [2]. The discovery of streptomycin antibiotics by Waksman and Schatz in 1943 marked the first effective anti‐TB drug and a significant milestone in anti‐TB therapy during the 1940s [3]. The current standard treatment for TB is effective but often cumbersome. Patients with drug‐susceptible MTB infections and mild cases must take standard antibiotics for 6 months [4]. These commonly used antibiotics, known as first‐line drugs (FLDs), include ethambutol (EMB), isoniazid (INH), rifampicin (RIF), and pyrazinamide (PYZ) [5]. If FLDs fail, second‐line drugs are used, which include levofloxacin, moxifloxacin, and bedaquiline [5].

Additionally, pretomanid—a new second‐line drug—was introduced in 2019 for treating drug‐resistant TB (DR‐TB). Unfortunately, compliance declines with time due to the lengthy duration of treatment as well as undesirable side effects, as described in Table 1, which results in irregular consumption and program failure. In many situations, a lack of adequate healthcare infrastructure and an antibiotic shortage can encourage the development of resistance [14]. TB resistant to INH and RIF is known as MDR (multidrug‐resistant)‐TB, whereas MDR‐TB with additional resistance to an aminoglycoside and a fluoroquinolone is XDR (extremely drug resistance)‐TB [15].

**Table 1:** Common and serious adverse effects, frequency, management strategies, and precautions for each of the key anti‐TB drugs.

Anti‐TB drugs	Common side effects	Serious side effects	Frequency	Management	Precautions	References
Isoniazid (INH)	Nausea, vomiting, rash, fever	Hepatitis, peripheral neuropathy, seizures	Common	Vitamin B6 supplementation, liver monitoring	Liver disease, alcohol use, pre‐existing neuropathy	[6]
Rifampicin (RIF)	GI upset, rash, liver toxicity, reddish‐orange urine	Hepatitis, thrombocytopenia, renal impairment	Common	Liver function tests, hydration	Avoid alcohol, contact lenses, and birth control pills	[7]
Pyrazinamide (PYZ)	Arthralgia, nausea, vomiting, rash	Hepatotoxicity, hyperuricemia, gout	Common	Joint pain management, hydration	Liver disease, gout history	[8]
Ethambutol (EMB)	Optic neuritis, nausea, vomiting, headache	Loss of vision (rare, reversible with discontinuation)	Less common	Regular eye exams	Regular vision tests, pre‐existing eye conditions	[9]
Streptomycin (STM)	Pain at the injection site, hearing loss, nausea	Ototoxicity, nephrotoxicity, vertigo	Less common	Monitor kidney and hearing function	Kidney disease, pre‐existing hearing impairment	[10]
Capreomycin (CAP)	Pain at the injection site, fever, nausea, rash	Nephrotoxicity, ototoxicity	Less common	Kidney and hearing function monitoring	Kidney disease, hearing issues	[11]
Clofazimine (CFZ)	Skin discoloration, GI upset, dry skin	Corneal pigmentation, hepatotoxicity	Less common	Symptomatic treatment, liver monitoring	Pre‐existing liver disease, photo sensitivity	[12]
Linezolid (LZD)	Nausea, diarrhea, headache, dizziness	Myelosuppression, peripheral neuropathy	Less common	Blood count monitoring	Blood disorders, long‐term use	[13]

TB and DR‐TB have seen great advancements in research; however, most of the data regarding DR‐TB focuses on the use of anti‐TB medications as the main backbone of therapy, and isolated advancements have been made in methods of drug delivery. There is little overlap in the literature discussing how different emerging multidisciplinary strategies can complement each other in treating DR‐TB [16]. Overall, there is a significant need for a unified approach to connect emerging nanotechnology‐based methods of drug delivery with host‐directed treatments (HDTs) and natural products; this approach should also include the importance of theranostics for improved TB diagnosis and ongoing treatment monitoring [17]. This focused review provides an overview of advanced TB therapies, along with nanocarrier‐based drug delivery, host‐directed strategies, and bioactive natural products for TB. Additionally, the emerging role of theranostic platforms to achieve targeted, effective, and personalized management of TB has also been discussed. Bridging these separate domains leads to a new perspective for overcoming issues related to DR‐TB and treatment ineffectiveness, and delays in diagnosis related to TB [18].

### 1.1. Rising Drug Resistance in TB

The current standard treatment of TB is efficient for drug‐susceptible MTB infections. Individuals with drug‐susceptible TB (DS‐TB) without complications must take multiple antibiotics for 6 months. Due to compliance being inconsistent, the WHO recommends Directly Observed Treatment, Short‐course (DOTS) and supervised treatment, adding significant infrastructure to an unusually prolonged treatment regimen. The rates of treatment failure have increased along with toxicity and significantly more expensive medicines due to the rise in drug resistance and its management [4]. Table 2 describes the risk factors involved in drug resistance to TB. DR‐TB is becoming even more common, which is putting pressure on the scientific and medical communities to study alternative and more effective treatment options [19].

**Table 2:** Risk factors involved in drug resistance to TB.

Risk factors	Description
Limited access to healthcare	Restricted access to diagnostics, medication, and follow‐up care exacerbates resistance.
Substance abuse (e.g., alcohol)	Alcohol and drug abuse can impair adherence to TB treatment, increasing resistance.
Malnutrition	Impaired immunity increases the likelihood of active TB and challenges treatment outcomes.
Poor drug quality	Use of substandard or counterfeit medications leads to incomplete bacterial eradication.
HIV coinfection	Immunocompromised states increase susceptibility to TB and resistance development.
Previous TB treatment	Patients with incomplete or unsuccessful prior treatment are at higher risk of resistance.
Delayed diagnosis	Prolonged undiagnosed cases allow disease progression and transmission of resistant strains.
Genomic factors	Natural mutations in TB genes like *katG*, *inhA*, *rpoB*, *pncA*, and *embB* confer resistance to first‐ and second‐line drugs.
Noncompliance of patients	Missed doses or treatment interruptions lead to incomplete bacterial eradication and resistance.
Inadequate treatment	Use of incorrect or suboptimal drug regimens, irregular intake, or premature discontinuation.

The WHO characterized drug resistance in several categories: (i) mono resistance (MR), which is resistant to one of the FLDs only; (ii) poly resistance (PR), which is resistant to many FLDs instead of INH and RIF; (iii) MDR, which is categorized as having resistance to at least INH and RIF; (iv) XDR, which is resistant to most second‐line injectable drugs (kanamycin [KAN], amikacin [AMK], and capreomycin), as well as fluoroquinolone‐resistant in addition to an MDR; and (v) rifampicin resistance (RR), which was identified phenotypically or genotypically, manifests with or without resistance to other anti‐TB medications. It includes resistance to all forms of RR, MDR, and XDR [4]. Pre‐XDR‐TB is resistance to RIF and any fluoroquinolone (second‐line anti‐TB drug), as further detailed in Table 3. However, globally, rates of DS‐TB and MDR‐TB have been gradually declining. MDR is seen in 3.4% of newly diagnosed TB and 18% of pretreated cases, and XDR‐TB is increasing, which makes management much more difficult. In 2018, XDR‐TB was identified in 6.2% of MDR cases [20]. In the world, 8% of TB patients have the condition known as INH‐resistant but RIF‐susceptible TB. Levofloxacin, RIF, INH, and EMB for 6 months were recommended by the WHO in 2018. Later, this was updated to a 6‐month regimen of RIF, EMB, PYZ, and levofloxacin, with removal of INH once resistance is confirmed [20].

**Table 3:** Categories of drug‐resistant tuberculosis based on resistance patterns.

Type of TB	Definition
Isoniazid (INH)‐resistant TB (Hr‐TB)	Resistance to INH with susceptibility to rifampicin (RIF)
RIF‐resistant TB (RR‐TB)	Resistance to RIF, with or without resistance to other first‐line drugs
Multidrug‐resistant TB (MDR‐TB)	Resistance to at least both INH and RIF
Pre‐extensively drug‐resistant TB (pre‐XDR‐TB)	MDR/RR‐TB with additional resistance to any fluoroquinolone
Extensively drug‐resistant TB (XDR‐TB)	MDR/RR‐TB with resistance to any fluoroquinolone and at least one additional Group A drug (e.g., bedaquiline or linezolid)

### 1.2. Factors Responsible for Drug Resistance and Possible Combating Strategies

The development of DS‐TB has been linked to many factors related to management, healthcare providers, and patients (Table 4). These include weak TB control programs leading to improper supply of efficient treatments, limited patient retention, use of weaker treatments, inconsistent and insufficient drug supplies, and inadequate supervision. Contributing factors also include healthcare workers′ lack of knowledge about TB, improper prescription practices, treatment pauses due to adverse impacts, patients′ noncompliance with treatment, over‐the‐counter anti‐TB medications available without proper prescription, patients′ illiteracy rate, coinfection with HIV and diabetes, delayed diagnosis, as well as drug‐susceptible testing of MTB isolates, poor quality drug formulations, lack of quality control measurements, and misuse of anti‐TB drugs for treating conditions unrelated to TB [25]. The use of drugs eventually contributes to drug resistance. However, it could be effectively managed through accurate diagnosis and personalized or tailored treatment regimens for individual patients. Among the significant health challenges of the 21st century, the rise of antimicrobial resistance (AMR) is a serious challenge to public health worldwide. The global proliferation of DR pathogens prompted the WHO to compile a priority list of high‐risk bacterial pathogens [1]. Notably, DR‐TB accounts for over 29% of global deaths associated with AMR, designating MTB as a high‐priority pathogen. In response, collaborative efforts among academic institutions, non‐profit organizations, and research and development (R&D) sectors have intensified, leading to funding initiatives that aim to combat the DR‐TB threat. Consequently, advanced research and drug development efforts are underway to eradicate MTB effectively [26].

**Table 4:** Factors linked with anti‐TB treatment leading to drug resistance and strategies combating drug resistance.

Factors leading to drug resistance	Description	Strategies combating resistance	Description of method	References
Improper use of antibiotics	Inadequate or incorrect use of TB drugs (e.g., stopping treatment early, incorrect dosage, or using the wrong drug regimen)	Directly Observed Treatment, Short‐course (DOTS)	Ensures patients adhere to prescribed treatment by observing them take their medication, reducing the chances of resistance	[21]
Incomplete treatment course	Not completing the full course of treatment can allow surviving bacteria to develop resistance.	Extended treatment regimens	Longer and more rigorous treatment plans for patients to ensure full eradication of the bacteria	[22]
Inadequate drug supply	Shortages or inconsistent availability of TB medications can lead to the use of ineffective alternatives or incomplete treatment.	Improved access to TB medications	Ensuring a steady and reliable supply of the correct medications in both rural and urban areas	[23]
Transmission of drug‐resistant strains	Spread of drug‐resistant TB strains from one person to another, especially in crowded or poorly ventilated areas	Screening and isolation	Early diagnosis and isolation of infected individuals, especially those with drug‐resistant TB, to prevent further transmission	[24]

### 1.3. Mechanism and Transmission of Drug Resistance

There are several ways that bacteria become resistant to antibiotics. One such strategy is the synthesis of enzymes that break down or render antibiotics inactive, thus reducing their impact on the bacterial cell. Enzyme *β*‐lactamase breaks down penicillin and other modern *β*‐lactam antibiotics, which are present in MTB encoded by the *blaC* gene/Rv2068c [27]. Moreover, changes in the bacterial cell wall structure can impede the entry of antibiotics, limiting their ability to reach the intended target. Collectively, these resistance mechanisms enable microorganisms to survive and proliferate despite antibiotic treatment, significantly complicating infection management and control efforts [28].

**Figure 1:**
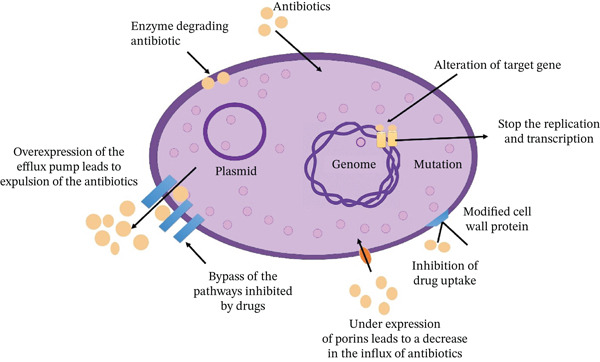
Various mechanisms of antibiotic resistance in MTB.

DR strains of MTB were thought to be less contagious than drug‐susceptible strains for several years, but recent research has shown that they may severely infect a person exposed to them. It takes at least 10–12 weeks for isolation, identification, and drug susceptibility testing using classical techniques [29]. Figure 1 explains various mechanisms involved in MTB resistance. Despite being the most common, the prolonged waiting time for results could postpone the start of appropriate treatment, which could lead to the patient spreading drug‐resistant infections throughout the community. Direct sensitivity testing saves at least 4 weeks for determining the resistance status. However, this approach is not very helpful in paucibacillary and smear‐negative specimens. The period between specimen collection and outcomes is now 2–3 weeks or less due to several more recent techniques, such as molecular diagnostics. However, these techniques necessitate significant technical infrastructure. These techniques place financial limits on a standard laboratory setup in impoverished countries and call for high technical competence [29].

### 1.4. Major Resistances Usually Due to Mutations

A wide range of mutations in the mycobacterial genome have surfaced that render these antibiotics ineffective and the organisms resistant, thereby adding to the challenges of TB treatment. WHO released a list of 17,000 MTB mutations and their correlation with resistance [30]. The resistance breakout makes disease management and treatment more difficult, particularly for patients infected with HIV [1].

### 1.5. Mutation in Genes Linked With FLDs

RIF eliminates MTB by linking the beta subunit of RNA polymerase, halting the elongation of the RNA chain and disrupting the transcription process [28]. The subunits of RNA polymerase *α*, *β*, *β*  ^′^, and *σ*, encoded by the *rpoA, rpoB*, and *rpoC* genes, respectively, have undergone extensive conservation. At its unique 81‐base pair hot spot region, commonly known as the RR determining region, the codon 426‐452 of the *rpoB* gene is mutated, which leads to RIF ineffectiveness [31]. Resistance to INH has been associated with mutations in the *katG*, *ahpC*, and *inhA* genes. The catalase/peroxidase enzyme, encoded by the *katG* gene, must be active for the mechanism of action of INH [32]. EMB prevents the biosynthesis of arabinogalactan by blocking the arabinosyltransferase in the cell wall. The EMB resistance is caused by the overexpression of the *emb* gene or a mutation in its protein structure, both of which reduce the drug′s effectiveness by overcoming its antibacterial effects. A mutation in the *embB* gene at Position 306 could represent another explanation of the EMB resistance, which is caused by the substitution of methionine with leucine or isoleucine [33]. The resistance in PYZ is often caused by a mutation in the *pncA* gene at the putative promoter′s 561 and 82 bases [34]. The 16S rRNA undergoes destabilization through the mutation of the *rpsL* gene, which causes streptomycin resistance. The structure of 16S rRNA is stabilized by S12 ribosomal protein, which acts as a stabilizing factor for 16S rRNA, ensuring the ribosome′s integrity and functionality during protein synthesis [35].

### 1.6. Mutation in Genes Linked With SLDs

In recent research, 20% of 200 MDR strains tested positive for ofloxacin (OFX), 1.5% tested positive for AMK, and 2.5% for KAN. Also, *gyrA*, *gyrB*, *rrs*, and *tlyA* gene mutations were observed in 14.5% of those MDR strains. One of the most predominant mutations was found in QRDR of *gyrA* and *gyrB* genes, which had an Ala90Val mutation in *gyrA,* which was present in 67.5% of the 40 OFX‐resistant isolates. Out of those 200 MDR isolates, only 25% were resistant to KAN and AMK, which showed A1401G or Lys89Glu mutations, whereas the remaining 8 had *rrs* and *tlyA* gene mutations [36].

Another study conducted in 2016 examined mutations in genes linked with SLDs in MTB isolates, revealing significant differences in mutations between monoresistant strains and cross‐resistant strains. The G878A mutation in the *rrs* gene was identified as a novel mutation that has been associated with mono‐ and cross‐resistance to AMK, KAN, and CAP. Additionally, its insertion in molecular assays has shown the potential to improve CAP resistance detection sensitivity [37]. The specific linkage or genetic subgroup of a species or population based on genomic data is known as the Euro‐American X3 lineage, and it is associated with this mutation. Strains with mutations in the efflux pump genes Rv1258c and Rv0194 were found to be cross‐resistant to CAP, KAN, and AMK [27].

The resistance in CAP was associated with a mutation in the *rrs* and *tlyA* genes. In the KAN‐susceptible isolates, the Eis C12T mutation was found in one isolate with an AMK‐KAN‐CAP cross‐resistant strain. The genotype MTBDRsl assay identified the A1401G mutation in cases of AMK‐KAN‐CAP and AMK‐KAN‐resistant clinical isolates. A combination of genotype MTBDRsl and DNA sequencing uncovered 72 gene mutations associated with resistance to SLIDs [38]. A list of various genes involved in anti‐TB drug resistance is shown in Table 5.

**Table 5:** Gene mutations lead to alteration of the product and mechanisms of action, which contribute to TB drug resistance.

Drug (year of discovery)	MIC (*μ*g/mL)	Gene involved in resistance	Gene function	Mechanism of action	Mutation frequency (%)	References
Isoniazid (1952)	0.02–0.2	*katG, inhA*	Catalase‐peroxidase, enoyl ACP reductase	Inhibition of mycolic acid synthesis and other multiple effects	50–95, 8–43	[28]
Pyrazinamide (1952)	16–100	*pncA, rpsA, panD*	Pyrazinamidase/ribosomal S1 protein	Depletion of membrane energy, inhibition of trans‐translation, pantothenate, and CoA synthesis	72–99	[39]
Rifampicin (1966)	0.05–1	*rpoB*	*β*‐subunit of RNA polymerase	Inhibition of RNA synthesis	95	[28]
Ethambutol (1961)	1–5	*embB, ubiA*	Arabinosyl transferase, DPPR synthase	Inhibition of arabinogalactan synthesis	47–65	[40]
Streptomycin (1943)	2–8	*rpsL, rrs, gidB*	S12 ribosomal protein, 16S rRNA	Inhibition of protein synthesis	52–59, 8–21	[27, 39]
Amikacin/kanamycin (1957)	2–4	*rrs, eis, whiB7*	16S rRNA, transcriptional regulator	Inhibition of protein synthesis	76	[27]
Capreomycin (1960)	2–4	*rrs, tlyA*	16S rRNA, 2′‐O‐methyltransferase	Inhibition of protein synthesis	85	[27]
Quinolones (1963)	0.5–2.5	*gyrA, gyrB*	DNA gyrase subunits A and B	Inhibition of DNA synthesis	75–94	[40]
Ethionamide (1956)	2.5–10	*etaA/ethA, ethR, inhA*	Flavin monooxygenase, enoyl ACP reductase	Inhibition of mycolic acid synthesis	37, 56	[41]
PAS (1946)	1–8	*thyA, dfrA, folC, ribD*	Thymidylate synthase, dihydrofolate reductase	Inhibition of folic acid and thymine nucleotide metabolism	37	[42]
Cycloserine (1955)	10–40	*alr, ddl, cycA*	Alanine racemase, D‐alanine ligase	Inhibition of cell wall peptidoglycan synthesis	To be determined	[43]
Bedaquiline	0.06	*atpE*	ATP synthase c subunit	Inhibition of mycobacterial ATP synthase	Low	[44]
Pretomanid (PA‐824)	0.015–0.25	*ddn, fgd1, fbiC*	Deazaflavin‐dependent nitroreductase	Generation of reactive nitrogen species disrupting cell metabolism	Low	[7]
Delamanid (OPC‐67683)	0.006–0.024	*ddn*	Deazaflavin‐dependent nitroreductase	Inhibition of mycolic acid biosynthesis	Low	[45]
SQ109	0.1–1	*mmpL3*	Mycobacterial membrane protein	Inhibition of cell wall biosynthesis	Low	[46]
Linezolid	0.25–2	*rplC, rrl*	50S ribosomal subunit	Inhibition of protein synthesis	3–11	[47]
Clofazimine	0.031–0.25	*rv0678, atpE*	Transcriptional repressor, ATP synthase c subunit	Generation of reactive oxygen species, inhibition of ATP synthase	10–35	[48]

### 1.7. Diagnostics and Current Advancements

TB infection can be caused by MTB, which is influenced by multiple risk factors that increase host susceptibility, as described in Table 6. Early and accurate diagnosis of TB is essential for effective disease management and prevention of transmission. Conventional methods such as sputum smear microscopy and culture remain widely used; however, they are limited by low sensitivity and long turnaround times [48]. The term “bacteriologically confirmed” refers to instances of TB that have been recognized using culture, rapid molecular testing recommended by WHO, sputum smear microscopy, or lateral flow urine lipoarabinomannan (LF‐LAM) tests. The microbiological detection of TB is crucial for the precise diagnosis of patients and initiating the most effective treatment strategy as soon as possible. “Clinically diagnosed” refers to TB patients diagnosed without bacteriological evidence [48]. Recent advancements in molecular diagnostics, including nucleic acid amplification tests (NAATs), GeneXpert, and CRISPR‐based detection systems, have significantly improved diagnostic accuracy and rapid detection of drug resistance. However, their high cost and infrastructure requirements limit widespread implementation in resource‐limited settings [49]. With new and advanced techniques such as CRISPR, AI (artificial intelligence)‐assisted detection, and Next Generation Sequencing (NGS), the TB diagnosis and its treatment have been significantly improved, especially in limited resource settings. CRISPR is quick, affordable, and accurate for the diagnosis of TB. One such example is the “TB one‐pot” assay, which precisely detects TB from other clinical samples by cross‐priming amplification with CRISPR/Cas12b detection in a solo reaction [50]. Complete genomic analysis of MTB is shortened by NGS, which helps in identifying specific gene markers that affect resistance to drugs and supports the development of treatment plans. For effective management of TB in low‐resource settings, affordable and movable sequencing techniques are being developed, as NGS techniques could be very expensive [51]. In the present situation of the world, where resources are limited, CRISPR, NGS, and AI‐assisted detection showed wide potential in refining the diagnosis of TB and also its treatment. To eliminate TB, countless R&D efforts have been made, which are both affordable and accessible [52].

**Table 6:** Risk factors contributing to tuberculosis infection and resistance.

Risk Factors	Description
MTB resistance	The causative agent of TB can develop resistance to drugs. This includes as follows:
Multidrug‐resistant TB (MDR‐TB)	Resistance to at least two first‐line drugs: INH and RIF
Extensively drug‐resistant TB (XDR‐TB)	Resistance to MDR‐TB plus resistance to any fluoroquinolone and at least 1‐s‐line injectable drug, bedaquiline or linezolid
Totally drug‐resistant TB (TDR‐TB)	Resistance to all drugs used to treat TB is extremely rare.
Host resistance factors	
Immune response	A strong immune response can limit the progression of TB. Inherited factors can influence susceptibility, including genetic polymorphisms.
HIV coinfection	HIV‐positive individuals are more susceptible to TB due to immune suppression.
Nutritional status	Poor nutrition can weaken the immune system, making it harder to fight TB.
Age and gender	Children and older adults have weaker immune responses, increasing TB susceptibility.
Environmental factors	
Crowded living conditions	Overcrowding, poor ventilation, and inadequate sanitation contribute to the spread of TB.
Inadequate healthcare access	Limited access to healthcare and diagnostic services increases the likelihood of undiagnosed and untreated TB, leading to drug resistance.
Treatment‐related factors	
Nonadherence to treatment	Failure to complete a full course of TB treatment can contribute to drug resistance.
Inappropriate use of drugs	Incorrect or incomplete treatment regimens can result in resistance developing.
Delay in diagnosis	Delayed diagnosis and treatment may allow the bacterium to develop resistance, especially in drug‐sensitive cases.

### 1.8. Standard Treatment for TB

RIF remains a cornerstone of TB therapy due to its inhibition of bacterial RNA polymerase [53]. RIF is the key antibiotic in TB treatment due to its ability to inhibit bacterial RNA polymerase, thereby preventing mRNA synthesis and exerting a bactericidal effect on MTB [54]. The 1980s had seen the establishment of the now‐prescribed regimen of RIF, INH, EMB, STM, and PYZ due to the findings from successive trials lasting 6 months, as shown in Table 6. Standard TB treatment involves a multidrug regimen consisting of RIF, INH, PYZ, and EMB. Although this regimen is effective for DS‐TB, the emergence of MDR‐ and XDR‐TB has significantly reduced treatment success rates, requiring prolonged, toxic, and less effective second‐line therapies. These limitations highlight the urgent need for improved therapeutic strategies [30]. A combination of FLDs (undergo antibiotic susceptibility testing) is used to treat MDR‐TB and XDR‐TB. When compared to the DS‐TB regimen, MDR and XDR‐TB treatment typically takes longer, is less successful, more expensive, and requires injectable medications. Even when given directly monitored treatment, the percentage of MDR‐TB patients who were cured in a retrospective cohort analysis was not higher than 69% for treating DR‐TB. These standard medications have various side effects on the human body. New suggestive molecules and formulations with innovative mechanisms and fewer side effects must be explored [55].

### 1.9. Current Status of Treatment for DR‐TB

The standard first‐line chemotherapy regimen for TB is summarized in Table 7. A considerable number of drug candidates and recently licensed medications with new mechanisms have made it possible to develop new treatments to treat DR‐TB, MDR, and XDR‐TB infections [56]. A significant change since 2018 was that all MDR‐TB/RR‐TB cases were treated with oral drugs, and injectables were removed from the prior regimens [57]. Injectable aminoglycosides, associated with serious side effects, including hearing loss, are not used in these shorter regimens. In 2022, three major protocols were included in the guidelines for the treatment of DR‐TB: First is the combination of bedaquiline, pretomanid, linezolid, and moxifloxacin (BPaLM), which are the first four drugs in a short 6‐month MDR‐TB/RR‐TB course that can be extended for another 3 months; for patients with pre‐XDR‐TB, moxifloxacin can be excluded, thus referred to as BPaL [14]. Second is the all‐oral short regimen of 9 months for patients with MDR‐TB/RR‐TB (extendable by 2 months if required). Third is a longer course of 18–20 months that may include an injectable drug such as AMK. Priority is given to the first protocol and recommended for people 14 years or older with MDR‐TB/RR‐TB/pre‐XDR‐TB. The New Investigational Drugs for XDR‐TB (NIX‐TB) trial evaluated a 6‐month course (NCT02333799) of bedaquiline, pretomanid, and linezolid, giving 90% success in a small number of patient groups diagnosed with MDR‐TB and XDR‐TB [14]. However, adverse events such as peripheral neuropathy were noted in patients receiving 1200 mg of linezolid daily. In a trial conducted by ZeNIX (ClinicalTrials.gov number, NCT03086486), the efficacy of different doses of linezolid in a 3‐drug regimen was examined [58]. A dose of 600 mg linezolid daily showed a favorable outcome during the 6‐month course with reduced side effects, lowering the toxicity and length of the treatment, which is an important step in enhancing patient outcomes [48, 59].

**Table 7:** The standard chemotherapy for TB treatment.

Drug	Category	Dosage	Duration	Additional notes	References
(INH)	First‐line drug (bactericidal)	5 mg/kg (max 300 mg) daily	6 months (initial phase: 2 months)	Monitored for hepatotoxicity, peripheral neuropathy	[48]
(RIF)	First‐line drug (bactericidal)	10 mg/kg (max 600 mg) daily	6 months (initial phase: 2 months)	Watch for drug interactions, liver function	[48]
(PYZ)	First‐line drug (bactericidal)	25 mg/kg (max 2000 mg) daily	2 months (initial phase)	Monitor for hepatotoxicity, hyperuricemia	[48]
(EMB)	First‐line drug (bacteriostatic)	15 mg/kg daily	2 months (initial phase)	Monitor for optic neuritis, visual acuity	[48]
(STM)	Second‐line drug (injectable)	15 mg/kg daily	2 months (initial phase)	Renal and ototoxicity monitoring	[48]

With the approval of bedaquiline, delamanid, and pretomanid, TB treatment has been transformed to another level, with a motivation for TB drug development. Bedaquiline, approved in 2012 (sold with the brand name Sirturo), is a diarylquinoline that inhibits mycobacterial ATP synthase [43]. The FDA authorized delamanid in 2014 and has been approved in cases of MDR and RR‐TB. Pretomanid, a 6‐nitro‐2,3‐dihydro‐imidazo‐oxazole, works by disrupting the cell wall as it inhibits the synthesis of mycolic acid. Additionally, it inhibits non‐replicating bacteria in anaerobic environments after the release of nitric oxide [60]. Pretomanid was recently approved in 2019 for XDR‐TB and has demonstrated in vivo activity in animal TB models [61]. Linezolid, an oxazolidinone inhibitor of bacterial protein synthesis, has been used extensively worldwide against drug‐resistant, Gram‐positive bacterial infections [62]. However, resistance to linezolid has been reported in MDR‐TB cases, mainly from China and Turkey. Many adverse events were related to the treatment of MDR‐TB cases with only linezolid, and it was recommended that improved safety and efficacy can be seen in combination with bedaquiline and delamanid [63]. A new prototype for developing a PAN‐TB treatment has become more popular in the last 5–10 years because of an expanding pipeline for anti‐TB medication candidates [4].

Because there is a paucity of highly effective anti‐TB medications, HDT may be a tempting complementary strategy to boost or restore host immunity [64]. HDTs contain drugs that fight MTB but are not microbicidal; instead, they modify host immunity and may either contribute to it or collaborate with anti‐TB medications to increase their efficacy [40]. Numerous clinical trials have shown that host‐directed adjunctive therapies with new mechanisms are likely to decrease the treatment duration for MDR‐TB, reduce transmission, and enhance patient outcomes. The majority of HDTs target host signaling pathways that are highly conserved, eliminating the risk of developing tolerance [65]. Several groups have been working on developing inhaled antibiotics to create novel and more efficient treatments for both susceptible and resistant MTB infections [66]. Numerous studies have shown that alveolar macrophages easily absorb inhaled particles or vesicles and that inhaled medications could enhance anti‐TB activity by raising the medicine concentration in granulomas and infected cells. This method of uptake may improve drug efficacy and treatment outcomes [67]. Clofazimine, azithromycin, ciprofloxacin, and nitric oxide gas are inhaled therapies for treating TB [68].

Despite the advantages and disadvantages of these chemically synthesized drugs, natural products play a significant role in potentially treating acute TB infections [69]. Two new anti‐TB antibiotics were discovered by Igarashi et al. [70], which are obtained from actinomycete metabolites: tubelactomicin A, caprazamycin, and CPZEN‐45, a semisynthetic antibiotic derived from caprazamycins [71]. Tubelactomicin A disrupts the production of mycolic acid, an essential lipid content of the MTB cell wall, which leads to cell wall destabilization. At the same time, caprazamycins exhibit potent activity against MTB by disrupting cell wall synthesis and eventually causing cell death, and CPZEN‐45 targets critical enzymes involved in cell wall synthesis, offering the potential for treating resistant strains of TB [70–72]. Despite these advancements, some countries continue to employ antiquated injectable regimens, often due to logistical challenges or limited availability of more modern drugs. To encourage the widespread use of BPaL and BPaLM, WHO and other international health partners intend to increase drug availability, educate medical professionals, and ensure cost‐effectiveness. These regimens are expected to significantly improve DR‐TB treatment outcomes globally [56].

### 1.10. Emerging Therapeutics in TB

#### 1.10.1. Host‐Directed Therapies

HDTs are an approach to boost the immune system, not just by attacking MTB with standard antibiotics [49]. Instead of killing the bacteria or preventing them from multiplying, HDTs work by affecting how cells behave, by tapping into cellular pathways such as (1) autophagy, (2) apoptosis, (3) inflammation, and (4) changes to how your cells metabolize food. Therefore, the use of HDTs for TB should result in a lower chance of resistance to antibiotics than using conventional antibiotics because HDTs do not create an environment of selective pressure on MTB [73]. Scientific literature has revealed that certain medicines (metformin, statins, and vitamin D) can enhance the functions of macrophages in the body to help face the challenging problems of MDR and XDR‐TB [65]. New developments have enhanced the immune function of individuals with TB, particularly through the involvement of host immune checkpoints (such as TNF‐*α*, IL‐1*β*, IFN‐*γ*) [74]. Additionally, more researchers are exploring new ways to combine host immune therapies with standard TB treatments to increase the effectiveness and decrease the time needed to treat TB [75].

#### 1.10.2. Natural Products as Anti‐TB Agents

Natural products are an important source for discovering new drugs to treat TB because of their diverse variety of structures and a wide range of different targets [76]. Natural products have many phytochemicals that can provide potential new treatments for TB through inhibition of mycobacterial cell wall synthesis, breakdown of the mycobacterial membranes, inhibition of efflux pumps, and modulation of the immune response of the host [77]. Through recent studies on plants, many small molecules that target essential mycobacterial enzymes (DprE1, InhA, ATP synthase) have been identified. Tubelactomicin A and derivatives of caprazamycin, both of which are derived from actinomycetes, work very well against drug‐resistant strains of TB [78]. Many natural products have immune‐modulatory properties that could be helpful as part of a host‐directed strategy for reducing inflammation‐related injuries to the lungs. Bioactive natural scaffolds continue to be a leading source of inspiration and guidance for new therapies or combination therapies for TB [79].

#### 1.10.3. Nano‐Based Drug Delivery for TB Treatment

**Figure 2:**
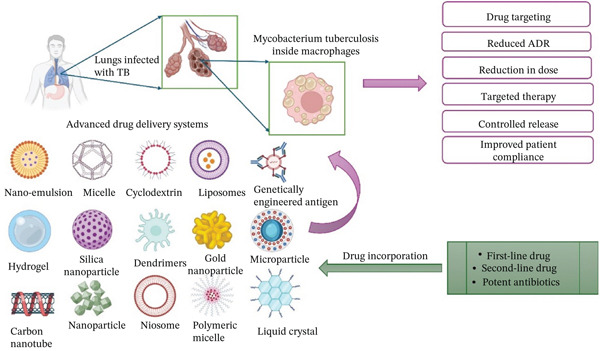
Different drug delivery methods for the treatment of TB. Nanocarriers permit controlled drug release and dose reduction for TB antibiotics that improve targeted therapy, lower adverse drug responses, and increase patient compliance.

Several innovative new ideas and approaches are being offered with nanotechnology and nanoparticle research, and may prove to be useful (Figure 2) because of enhancing TB treatment [80, 81]. Silver nanoparticle anti‐TB therapy proves promising to break through the barriers put on treatment by DR‐TB, which is commonly reported for most current synthetic antibiotics. To successfully implement AgNP anti‐TB treatment, many significant therapeutic issues must be addressed: insufficient delivery, variability in intramacrophagic antimycobacterial efficacy, and residual toxicity [82]. An ideal TB drug formulation showed a 12‐h retention time in simulated lung fluid, a mass median aerodynamic diameter (MMAD) of 3.71 *μ*m, and an overall particle size of 79.70 nm, permitting deep lung contact [83]. Most nanotechnology‐based drug delivery systems (DDSs) are composed of various compositions with unique structural and functional characteristics [84]. To lower the risks of traditional DDS, they can be easily modified by altering the components of the medication, polymers, excipients, and other additives used in their composition [85]. Nanoparticles comprise biocompatible and biodegradable components— including components like polymers, which can be natural, synthetic, or solid lipids [86].

A clinical trial used multiwalled carbon nanotubes (MWCNTs) to combine INH and fluoxetine (FLX), enhancing antibacterial activity through synergistic effects. Although findings suggest serotonin receptor modulation may activate autophagy to combat TB, further research on underlying signaling pathways is needed [87]. Different nanotechnology‐based platforms are used for TB treatment.

Nanotechnology has drastically changed the way we can deliver drugs for treating TB. We can now effectively target the drugs with the correct kinetics, traffic them intracellularly, and provide improved pharmacokinetics [81]. All of these factors are critical in effectively treating intracellular pathogens, like MTB. By using nanocarriers, the therapeutic benefit of a TB drug is increased by improving macrophage‐specific uptake, allowing the drug to cross the barrier, allowing the drug to remain in the macrophage for a longer period of time and to have lower levels of systemic toxicity [88]. These are all problems with using conventional TB chemotherapy to treat patients. When using polymeric nanoparticles, by controlling the degradation kinetics of the polymers, drugs are released in a controlled degree over time, which allows the drugs to remain in the macrophage for a longer time to kill MTB [89].

#### 1.10.4. Solid Lipid Nanoparticles (SLNs)

SLNs possess a lipid core structure that enables them to deliver anti‐TB compounds more securely, readily, and deeply into the lungs for prolonged periods of time; therefore, SLNs may improve pulmonary stability, availability, and cellular entry of anti‐TB drugs [90]. SLNs are small in size and take advantage of their aerodynamic properties to facilitate the uptake with alveolar macrophages, whereas their lipid matrix generates controlled and sustained release, permitting therapeutic concentrations to be maintained in the infected macrophages and granulomatous lesions [91]. SLNs increase the efficiency of the lipid matrix to fuse with the host cell membranes, leading to an enhanced amount of the drug accumulating intracellularly while decreasing systemic distribution and toxicity. Studies recently reported favorable pharmacokinetics, retention, and antimycobacterial biology, suggesting that SLNs represent useful carriers for inhaled anti‐TB therapeutics [92].

## 2. Liposomes

Liposomal formulation of anti‐TB drugs has the potential to improve DDS through the use of phospholipid‐coated liposomes due to their ability to encapsulate both hydrophilic and hydrophobic anti‐TB therapeutic agents, They provide high biocompatibility for pulmonary macrophages as well as deliver pharmaceuticals directly into their cytoplasm [93]. The bilayer structure of liposomes allows liposomal membranes to fuse with the host′s alveolar macrophage cell membranes and thereby facilitate lysosomal trafficking within the pulmonary macrophages, resulting in enhanced antimicrobial actions against MTB intracellularly [94]. The enhanced and modern liposomal formulations demonstrate improved stability of TB medicines with less cytotoxicity toward the macrophages and improved macrophage‐targeting specificity, along with improved penetration into the granulomas [88]. Advanced liposomal formulations have demonstrated improved intracellular retention of these medicinal compounds throughout the duration of the study in DR‐TB [93].

## 3. Microspheres

Microspheres made from Poly (lactic‐co‐glycolic acid) or PLGA represent a unique means to obtain targeted, continuous, and controlled delivery of drugs via the pulmonary route for extensive periods of time at a therapeutic concentration within infected macrophages and the lung tissues [95]. Utilizing an organic biodegradable polymer as the matrix, PLGA‐based microspheres allow drugs to be slowly diffused from the polymer matrix and provide a controlled manner for releasing drugs from inside the polymer. It has been shown that this system allows prolonged intracellular availability of the anti‐TB agent while minimizing the number of doses that must be administered, thus effectively enhancing the treatment of DR‐TB by targeting specific areas with no systemic toxicity [96]. New studies and new platforms for biodegradable microparticles demonstrate a significant increase in the amount of drug retained in the lungs, pharmacokinetic stability of the drug, and targeted delivery of the drug to the lungs of resistant TB models [88].

## 4. Chitosan (CS)

As a natural, cationic polysaccharide with the properties of being mucoadhesive, biodegradable, and biocompatible, CS is an effective drug carrier for administering medications for pulmonary TB [97]. In addition, the positive charge on the surface of CS enhances the electrostatic interactions between it and the negatively charged membranes of TB bacteria and host cells, which helps deliver CS to mycobacteria and increase its uptake by macrophages for intracellular delivery [98]. The protective effect of CS on a drug from enzymatic degradation allows for prolonged retention of that drug in the lungs and sustains the antimicrobial action of that drug. Furthermore, new developments have demonstrated that using CS‐based nanocarriers increases the concentration of the drug in cells by facilitating drug delivery to those cells; therefore, it improves the bioavailability of these drugs and greatly enhances their efficacy against MTB when treating resistant forms of TB [99].

### 4.1. Metal Nanoparticles

Due to their electron transfer capabilities (redox activity), disruption of cell membranes (disruptive activity), and ability to generate reactive oxygen species (ROS), nanoparticles from silver or gold can act as both carriers of drugs and provide intrinsic antimicrobial properties [100]. However, when bacteria are exposed to antimicrobial activity through nanoparticles, there will be a substantial increase in the kill rate of these bacteria via oxidative stress, membrane damage, protein denaturation, and DNA damage. Also, improved bioavailability of the drug inside the cell will occur through the use of these nanoparticles [101]. Due to the size of nanoparticles, they are easily taken up by macrophages and accumulate within these cells, permitting nanoparticles to provide an additional effect when combined with conventional anti‐TB agents [88]. Studies show that the antimycobacterial activity of silver and gold nanoparticles is achieved through improved bactericidal activity against intracellular bacteria, disruption of the mycobacterial cell membrane, and enhancement of the anti‐TB drug activity in resistant strains of MTB [102].

### 4.2. Nanoemulsions and Nanosuspensions

Pharmaceutical formulations such as nanoemulsions or nanosuspensions increase drug solubility, stability, availability, and targeting by increasing drug levels in lymphatic fluid and enhancing the distribution of the drug to infected tissues and granulomas [103]. These nanoformulations promote enhanced permeability through biological membranes, increased drug delivery to cells, and sustained therapeutic levels of the drug at the infection site [104]. The smaller particle size allows efficient uptake by macrophages and effective transport along the lymphatic system, leading to improved localization of the drug within infected lungs. Many recent studies have demonstrated that one of the beneficial aspects of these formulations is their ability to enhance pharmacokinetics, drug retention, and therapeutic outcomes in patients with TB, particularly in cases where the TB strains are resistant to standard treatments [105].

### 4.3. Carbon Nanotubes

Carbon nanotube and hybrid nanocarrier systems have demonstrated high drug‐loading capacity and excellent ability to transport drugs to the interior of cells and deliver them to the cytosol of cells, providing increased availability for use as a therapeutic agent [106]. In addition, these systems are capable of facilitating direct transport to the cytosol of the cell, activating the autophagy pathway, and providing immune‐mediated clearance of bacteria, thus enhancing their therapeutic effectiveness against intracellular MTB [103]. Because of the unique structural characteristics of these systems, they are able to penetrate cells efficiently, remain in the cells for an extended period of time, and demonstrate enhanced antimicrobial activity in combination with traditional anti‐TB drugs [18]. Recent studies showed that the system improves bacterial clearance and improves the efficacy of DR‐TB treatment models via the activation of the autophagy pathway [107].

Collectively, modern nanocarrier systems offer multifunctional therapeutic platforms that integrate targeted delivery, immune modulation, intracellular trafficking, and controlled release, representing a next‐generation strategy for combating DR‐TB [108].

Bedaquiline is a highly effective drug against DR‐TB but with linked side effects and pharmacokinetic limitations that may affect treatment outcomes. Bedaquiline has been incorporated into nanocarrier systems, particularly lipid‐based nanoparticles, to enhance drug delivery and reduce systemic toxicity. This formulation demonstrated sustained drug release, improved pulmonary and intracellular delivery, enhanced compliance, and potential to overcome challenges related to DR‐TB. These advancements highlight the potential of nanocarrier‐based bedaquiline formulations to improve the therapeutic efficacy and treatment outcomes [109]. Despite the promising advantages of nanocarrier‐based systems, challenges such as large‐scale manufacturing, long‐term safety, and Barcelos approval remain significant barriers to clinical translation. Although nanotechnology addresses drug delivery limitations, complementary strategies such as HDTs further enhance treatment outcomes by modulating the host immune response [110].

### 4.4. Critical Evaluation and Translational Perspective of Nanotechnology‐Based TB Therapies

The use of DDS based on nanotechnology has been shown to provide significant advances toward improving treatment for TB. However, the actual availability of these technologies for use in humans is limited [111]. Most nanoformulations are at relatively early stages of development despite preclinical data to suggest that they could work as intended; challenges associated with scaling up manufacturing, reducing variability in product quality, and establishing their long‐term safety record have delayed their advancement into human clinical trials [112]. Additionally, from a safety perspective, there are potential risks associated with using nanocarriers, including cytotoxicity, generation of oxidative stress, and the presence of undesirably high concentrations of nanocarriers in nontarget tissues; these risks can vary depending on their physicochemical characteristics [92]. To mitigate these risks, it will be necessary to conduct thorough toxicological assessments and studies evaluating the biodistribution of nanocarriers so that they can be used with confidence in clinical settings. Nanocarrier systems can enhance bioavailability, deliver drugs directly to macrophages, and release drugs at a sustained rate compared to traditional anti‐TB therapies [113].

Nanocarriers will also allow for longer retention of drugs, improve drug stability, and increase drug concentrations at sites of infection—especially in the lungs. Nanocarrier formulations designed for inhalation also facilitate targeted drug delivery to the lungs, whereas several challenges, such as stability and pulmonary safety of aerosolized delivery mechanisms, need to be addressed prior to widespread use [114]. The continued clinical translation of nanocarrier‐based therapies is currently hampered by regulatory and manufacturing issues such as the lack of a standardized regulatory pathway, inherent batch‐to‐batch variability, and limitations on the scale‐up process [115].

### 4.5. Drug Repurposing in TB

With the increasing drug resistance and higher mortality rates, the need for new and advanced TB treatments is also increasing. One such way to develop drug treatments can be achieved by drug repurposing [116]. Drug repurposing means finding existing medications that may be effective for treating conditions other than the ones for which they were developed. Drug repurposing can overcome the challenges of antibiotic resistance and the development of novel antibacterial drugs. Drug repurposing has proved to be crucial in TB treatment by discovering anti‐TB properties such as linezolid, vancomycin, and celecoxib [42]. Many drugs have been repurposed to date for TB treatment, as shown in Table 8. Drug repurposing can lead to improved TB management and has been used in numerous trials to find working TB inhibitors. Structure‐based virtual screening and molecular docking have identified compounds that form effective binding interactions with the target enzyme DprE1 [46]. New platforms and targets against TB are being produced due to re‐evaluating previously found drugs and proven targets, increasing the possibility of developing operative drug regimens for drug usage over and over again [118].

**Table 8:** Repurposed drugs for TB treatment.

Drug	Original indication	Mechanism of action for TB	Advantages	Challenges	References
Linezolid	Antibiotic (Gram‐positive bacteria)	Blocks protein synthesis by attaching to the 23S rRNA within the 50S ribosomal unit	Effective against MDR and XDR strains	Bone marrow suppression, neuropathy	[27]
Vancomycin	Antibiotic (Gram‐positive bacteria)	Inhibits cell wall synthesis	Potential synergy with other TB drugs	Limited oral bioavailability, nephrotoxicity	[27]
Celecoxib	Anti‐inflammatory	Targets host immune modulation and inflammatory pathways	Adjuvant treatment to reduce TB‐associated inflammation	Gastrointestinal and cardiovascular risks	[27]
Nitazoxanide	Antiparasitic	Induce autophagy and disrupt metabolic pathways in MTB	Targets the intracellular survival mechanism	Requires further clinical verification	[42]
Imatinib	Anticancer (chronic myeloid leukemia)	Triggers autophagy pathways in macrophages	Enhances host‐directed therapies	High cost and potential off‐target effects	[27]
Sulfamethoxazole	Antibiotic (urinary tract infections)	Inhibits folate synthesis in MTB	Cost‐effective and widely available	Lower efficacy as monotherapy	[117]
Amoxicillin‐clavulanic acid	Antibiotic (broad spectrum)	Inhibits cell wall synthesis and beta‐lactamase activity	Broad‐spectrum activity	Limited data on long‐term TB use	[27]
Carbapenems	Antibiotic (severe bacterial infections)	Target cell wall synthesis	Effective against MDR strains	High cost and limited global availability	[42]

In a study by Lorente‐Torres et al. [119], novel approaches to drug repurposing were examined. They highlighted the application of high‐throughput screening and bioinformatics to find drugs that might be effective against resistant intracellular infections. Sharma et al. [120] examined the importance of proteomics and bioinformatics along with the repurposing of drugs in developing new strategies to fight DR‐TB. Singh et al. [121] investigated the polypharmacological techniques and identified FDA‐approved drugs as possible inhibitors of MTB. When studies are taken altogether, these findings show that repurposed drugs can offer quick, effective, and affordable ways to treat drug resistance [122].

Novel and much more advanced techniques, such as AI, are being employed for developing repurposed drugs. AI is being used to find existing drugs that can be used for TB treatment [123]. AI‐powered drug repurposing uses generative models, machine learning, network‐based strategies, and natural language processing to effectively and affordably develop new treatments for well‐known medicines [124].

### 4.6. Evolution of the TB Elimination Program

WHO initially announced to eliminate TB by 2025, but due to the emergence of COVID‐19, which caused a deceleration, it has now been shifted to 2035. WHO has executed many plans and programs to eradicate TB globally, the initial being the End TB Strategy in 2014, which aimed to reduce mortality rates by 95% and new cases by 90% from the year 2015 to 2035. This emphasized patient‐centric care, bold policies, intense research, support systems, and innovations [125]. Along with the WHO’s End TB Strategy, many national and international programs were introduced to eliminate TB. Another WHO program was the Stop TB Partnership, a global network of national and international organizations. It was founded in 2001 to eliminate TB. The Global Plan to End TB, which highlighted priority actions and budgetary requirements to eradicate TB, has undergone various modifications. The nations involved in the collaboration were India, South Africa, Nigeria, Russia, and China [126].

#### 4.6.1. Centers for Disease Control and Prevention (CDC) Tuberculosis Elimination Programs

To get closer to TB eradication, the US CDC has set priorities. These include targeted testing and treatment of individuals with latent TB infection by including communities that have a high burden of TB disease, quick detection of TB transmission through genotyping techniques, and quick response to outbreaks [127]. The coordinated worldwide effort to eradicate TB through all‐encompassing techniques that include prevention, diagnosis, treatment, and research in this direction.

Recently, the WHO reported that TB incidence cases increased in 2022 to 10.6 million from 10 million in 2020. The downward trend of cases that had been reported till 2020 has now seen a reversal. This reversal was an impact of the post‐COVID era as a result of disruptions. According to the WHO, in the year 2022, eight nations were identified for nearly two‐thirds of the global TB burden with India at 27%, Indonesia (10%), China (7.1%), the Philippines (7.0%), Pakistan (5.7%), Nigeria (4.5%), Bangladesh (3.6%), and the Democratic Republic of the Congo (3.0%). The National TB Elimination Program (NTEP) in India seeks to eliminate TB, and its policies closely mirror the WHO′s End TB Strategy. Both place a strong emphasis on prevention, efficient treatment, and early diagnosis for all. India has implemented treatment plans, like the inculcation of shorter MDR‐TB medications, and advanced diagnostics like GeneXpert, which are advised by the WHO. India′s consonance with WHO guidelines is reflected in its preventive efforts, behavior modification campaigns, digitalizing healthcare, and increased funding. India has made a substantial contribution to international TB control initiatives by fusing local adaptations with global frameworks [128]. According to a recent report, a 16% reduction in TB cases and an 18% reduction in death cases were observed since 2015.

**Figure 3:**
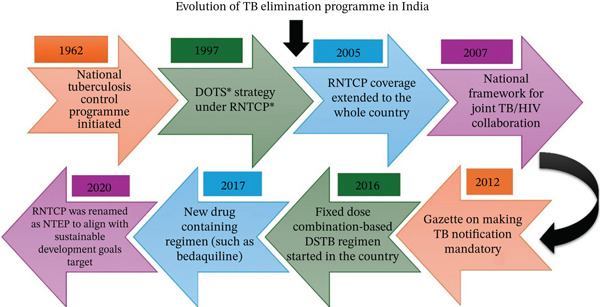
History and current scenario about the programs launched by the Indian Government for the eradication of TB/India′s TB Elimination Program. This graphic presents a timeline of the development of India′s TB Elimination Program. A sequence of linked, arrow‐shaped boxes, each color‐coded and grouped chronologically, serves as the visual representation of the chronology. Abbreviations: DOTS, Directly Observed Treatment, Short‐course; RNTCP, Revised National TB Control Program; NTEP, National TB Elimination Program.

The TB elimination program in India reflects a sustained and systematic approach to fight one of the nation′s greatest health problems. Figure 3 shows the basic diagnostic and treatment services from 1962 to the present day with the National TB Program. Recognizing its limitations and effectiveness, it transitioned to the Revised National Control Program in 1997, adopting the WHO′s DOTS strategy. With time, the program changed to include universal access to high‐quality diagnostic tools, free treatment, and DR‐TB management. In the year 2020, the RNTCP was rebranded as the NTEP, emphasizing the national government′s motive to end TB by 2025, 5 years prior to the global sustainable development goal timeline [129]. Despite significant progress, challenges such as stigma, MDR, and health system gaps remain, requiring sustained effort and collaboration to achieve elimination [129].

### 4.7. Challenges and Translational Considerations in Advanced TB Therapies

Although significant progress has been made in the fields of nanocarrier systems, HDTs, and natural products‐based therapies, numerous technical, commercial, and regulatory obstacles remain to successfully bring these innovations into adult health care settings [130]. Overcoming these barriers will be critically important to create a link from the laboratory to real‐life applications or solutions, particularly in high‐burden or low‐resource settings.

### 4.8. Cost and Economic Constraints

Advanced TB therapeutics face high costs for manufacturing, developing, and testing. Nanocarrier systems may need to be made with specialized materials, require high‐purity reagents, advanced instruments, and quality control, all of which drive up the cost of production [88]. The cost of large‐scale clinical trials to assess a new drug′s safety and efficacy also significantly increases development costs. This economic burden is particularly problematic because TB disproportionately affects low and middle‐income countries. The high cost of manufacturing might affect affordability, restrict government purchases, and limit integration into national TB control programs [131]. Therefore, it is important to identify cost‐effective formulation strategies, simplify synthesis methods, and work with public‐private partnerships to ensure equitable access to advanced TB therapeutics. Polymeric nanoparticles, inhalable nanocarrier formulations, and lipid nanoparticles usually require specialized manufacturing equipment, sterile processing methods, and extensive quality‐control testing. These requirements potentially increase production and expenses if compared to conventional oral anti‐TB medications [89, 132, 133]. As the TB cases majorly occur in low and middle‐income countries, the high cost of advanced therapeutics may confine large‐scale adoption within national TB control programs and healthcare systems [120, 134].

### 4.9. Scalability and Manufacturing Limitations

Nanobased DDS have shown particular preclinic advantages, including improved macrophage‐specific uptake, prolonged drug retention within pulmonary tissues, sustained drug release, improved pharmacokinetic profiles, and lower systemic toxicity compared with standard anti‐TB formulations. Despite this research, several challenges remain when translating these technologies to industrial‐scale manufacturing [88, 89, 132]. Achieving consistency between batches, uniformity in particle size, efficiency in loading drugs, and long‐term stability throughout the manufacturing process on a larger scale can be technically challenging [135].

In addition to the mentioned issues of consistent batch‐to‐batch performance, evaluation of the scalable nature of biodegradable polymers, lipid‐based systems, and hybrid nanocarriers will be necessary to ensure that they retain their ability to deliver therapeutic benefit, as well as their safety profile, once produced at the industrial level [136]. To attain regulatory approval for their manufactured products and remain commercially viable, companies must develop standardized formulation protocols and robust methodologies for scaling up manufacturing processes [135]. One of the major challenges in scaling nanomedicines is maintaining consistent particle size, drug loading efficiency, and release kinetics across production batches. For instance, slight changes during the synthesis of nanoparticles can significantly alter the drug bioavailability and therapeutic performance [89, 133]. Such batch‐to‐batch variability complicates regulatory approval and increases manufacturing expenses [120, 133].

### 4.10. Accessibility and Advanced Diagnostics

Rapid molecular diagnosis using NAATs, drug‐resistance profiling, and whole‐genome sequencing has become an increasingly important part of modern TB management, allowing for early diagnosis and precise therapy [137]. However, due to a variety of factors such as equipment cost, infrastructure, and the need for trained personnel, access to these advanced diagnostic technologies is still limited in many high‐burden countries. The lack of advanced diagnostics limits the ability to effectively deploy targeted therapies (nanocarrier‐based precision delivery systems) as well as HDTs that are tailored to the patient′s immune profile [138]. Therefore, treating patients effectively and monitoring resistance is very difficult without sufficient diagnostic support. In order to effectively implement advanced treatment strategies in clinical practice, we need to strengthen laboratory infrastructure, decentralize molecular testing platforms, and develop affordable point‐of‐care (POC) diagnostic tools [139]. Even though molecular platforms like GeneXpert, whole‐genome sequencing, and next‐generation sequencing provide rapid detection of TB, their implementation is limited by equipment costs, maintenance, and shortages of trained workers. Consequently, many high‐burden regions continue to rely on conventional diagnostic methods, delaying the start of appropriate treatment and limiting the effectiveness of personalized treatment methods [137, 128].

### 4.11. Optimization of DDSs

Nanocarriers and HDTs can both provide potential advantages for treatment; however, further optimization of these systems will be needed to improve their clinical efficacy [88]. Critical parameters such as drug loading capacity, release kinetics, targeting efficiency, immunogenicity, biodistribution, and long‐term toxicity must be evaluated before clinical use [140]. There are still challenges related to DDS, such as the delivery of the drug deep into the lung tissue where MTB resides, and also to avoid the rapid mucociliary clearance of these drugs from the airways by the body′s natural defense system. Furthermore, ensuring that adequate amounts of the drug penetrate the granulomatous lesions containing MTB is also necessary for therapeutic success [141]. The wide variability in patients′ immune responses will also affect the success of HDTs; therefore, personalized treatment should be explored. Additional comprehensive pharmacokinetic/pharmacodynamic studies are needed to identify optimal dosing regimens and improve therapeutic success [65].

### 4.12. Regulatory and Safety Considerations

There are currently evolving regulatory pathways and requirements for undertaking regulatory approvals for nanomedicines and HDTs combination regimens [120]. Generally, regulatory agencies require substantial information regarding safety, toxicity, pharmacokinetics, biodistribution, manufacturing quality, and efficacy of a treatment before granting a clinical trial approval. Despite the existence of broad regulatory requirements, specific guidelines related to TB nanomedicine formulations are limited [133]. Particularly, there are a few harmonized definitions related to assessing nanoparticle characteristics, long‐term biodistribution, safety in lungs, batch‐to‐batch uniformity for anti‐TB nanomedicines, and other developing products of nanotechnology. Concerns about the accumulation of nanoparticles in the body, unintended responses and side effects, and drug–drug interactions create a need for extensive preclinical and clinical testing before regulatory approval [89]. Both clearer and more uniform frameworks of regulation will enable the efficient clinical translation of approved TB therapies under development [89]. Various nanoformulations such as silver nanoparticles and carbon nanotubes have shown promise for their antimycobacterial activity; however, evaluation for long‐term biodistribution (tissue accumulation), oxidative stress, and potential toxicity from pulmonary exposure to humans remains incomplete [89, 133] These uncertainties related to safety create a barrier to timely regulatory evaluations and ultimately delay approval of nanomedicine therapies for clinical use [120, 133].

### 4.13. Future Perspectives

The future of management strategies for TB is increasingly turning toward technology integration and precision/personalized approaches. One area that shows great promise is developing individualized therapy for TB, where treatment plans are created tailored to an individual (e.g., using the person′s genome, immune profile, or susceptibility patterns of the drugs being administered) [142]. The utilization of an individualized approach to TB management has great potential for assisting in improving treatment outcomes while minimizing side effects and resistance development [143].

Implementation of AI and machine learning will create a new era in the management of TB; rapid prediction of drug resistance, optimization of treatment regimens, and early identification of potentially high‐risk patients are just a few ways these technologies will greatly enhance TB management efforts [144, 145]. The use of AI in conjunction with traditional methods of clinical, radiological, and genomic data will provide increased accuracy in diagnostic and therapeutic decision‐making. Owing to technological advancements in the areas of genomics, transcriptomics, proteomics, and metabolomics (collectively referred to as “multiomics”), researchers have gained further insight into the relationship between host and pathogen. This has resulted in the identification of new biomarkers and therapeutic targets for developing precision medicine strategies for the treatment of TB [146].

Additionally, TB vaccine development is being aided by the increasing availability of mRNA‐based vaccines and next‐generation vaccine platforms, which can be developed quickly and are highly specific. Both platform types provide the potential to stimulate a substantial immune response upon administration and therefore hold promise for future vaccine development [147].

In the area of translational nanomedicine, continued advancements in DDS are projected to result in the enhancement of targeted drug delivery, improved pharmacokinetics, and the development of theranostic approaches to enhance the potential for developing more efficient and personalized therapeutic approaches through the combination of nanotechnology with advanced diagnostic modalities and host‐directed therapeutics [148].

Implementing personalized TB therapy is quite challenging in many high‐burden regions due to inadequate access to advanced molecular diagnostic tools, genomic sequencing facilities, and specialized healthcare infrastructure [137, 139]. Therefore, an alternative pragmatic approach for implementation may be phased implementation strategies using the combination of lower‐cost molecular tests (GeneXpert and targeted drug resistance tests) and simplified clinical risk stratification tools. The use of regional reference labs, digital health platforms, and AI‐assisted decision support systems can extend precision medicine to resource‐limited areas without universal access to expensive sequencing technologies [128, 144, 145]. Continuous investments in lower‐cost POC diagnostic testing, laboratory capacity building, and integrating genomics and multiomics data into clinical workflows are critical to ensure that the benefits of personalized TB therapy can be realized in practice in high‐burden countries [142–144].

Future research must focus on advanced molecular diagnostics, like the expansion of rapid, affordable diagnostic tools leveraging whole‐genome sequencing and high‐end screening to identify drug‐resistant strains at an early stage and provide guidance for personalized treatments [137, 139, 142]. Nanotechnology‐based drug delivery approaches also prioritize the preclinical and clinical validation of nanocarrier systems to improve bioavailability, targeted drug delivery, and reduce toxicity [80, 81, 113]. HDTs also explore immune‐modulating therapies to complement existing antimicrobials, reducing reliance on prolonged antibiotic regimens [40, 64, 65]. Drug repurposing could accelerate clinical trials to validate the efficacy of repurposed drugs and combination therapies, particularly for MDR‐ and XDR‐TB cases [119, 121]. Achieving global scalability of these technologies requires robust collaborative efforts among academic institutions, pharmaceutical industries, and government agencies. Such partnerships can facilitate resource sharing, accelerate clinical trials, and address implementation challenges in resource‐limited settings. Coordinated initiatives, such as global funding for TB research and technology transfer, are vital to ensure equitable access to innovative diagnostics and treatments worldwide [1, 126, 131]. Through multidisciplinary collaboration and focused innovation, these advancements hold the potential to transform TB management, reduce global mortality, and pave the way toward TB elimination [1, 125, 126].

### 4.14. Knowledge Gaps and Future Research Priorities

Although considerable progress has been made with DDS based on nanotechnology, HDTs, drug repurposing, and antimycobacterial therapeutic agents derived from natural products, multiple critical areas of knowledge have not progressed. First, most evidence of efficacy from DDS, HDTs, drug repurposing, and natural products has been generated from in vitro or animal studies, with limited data being derived from larger clinical trials [93, 96, 132]. There are currently no standardized methods for determining long‐term safety, biodistribution, lung toxicity, or batch‐to‐batch reproducibility of DDS or HDTs, thus creating barriers to clinical translation and ultimately becoming regulatory approved [89, 120, 133]. Second, although there are indications that HDTs are useful in the development of immune‐mediated control mechanisms for MTB, little is known about the optimal target and length of treatment for HDTs or the criteria to select patients [40, 108, 112]. Additionally, although natural products provide excellent candidate compounds for the development of new drugs that will inhibit the growth of mycobacteria, there are many obstacles to the standardization, bioavailability, mechanistic evaluation, and clinical validation of natural products, leading to a lack of new drugs moving toward approval [42, 46, 118, 119]. Further, barriers to integrating AI, genome, and multiomic into the routine management of TB remain due to a lack of infrastructure, high cost of implementation, and limited access to advanced diagnostics in a number of high‐burden settings [139, 142, 145, 146].

Future research should prioritize large‐scale clinical validation of advanced therapeutic, development of standardized regulatory frameworks for nanomedicines, identification of predictive biomarkers for personalized therapy, and the creation of affordable POC diagnostic technologies suitable for resource‐limited settings [120, 133, 137, 139]. Greater emphasis on multidisciplinary collaborations among clinicians, microbiologists, nanotechnologists, computational scientists, and policymakers would be essential to accelerate the translation of emerging TB therapies from laboratory to clinical practice [128, 144, 145].

## 5. Conclusion

TB continues to pose a major global health challenge, particularly due to the emergence of MDR and XDR strains. This review highlights the mutational mechanisms of drug resistance, advancements in molecular diagnostics, novel therapeutic strategies, and innovative DDS. Although first‐line and second‐line drugs have made significant progress in TB management, their limitations—such as lengthy treatment durations, toxicity, and resistance development—necessitate urgent improvements. Promising approaches like nanotechnology‐based DDS (lipid nanoparticles and MWCNTs), HDTs, and drug repurposing strategies show potential for enhancing drug efficacy, reducing treatment duration, and addressing resistance. Integrating advanced diagnostics, nanotechnology‐based drug delivery, HDTs, and theranostic approaches offers a promising framework for improving TB management. However, addressing challenges related to cost, scalability, and regulatory approval is essential for successful clinical translation.

## Author Contributions

D.V. and D.S. conceived the idea and supervised, and S.G., J.S., and S.B.J.P. wrote the initial draft and prepared the figures. D.S. and D.V. extensively edited the manuscript.

## Funding

No funding was received for this manuscript.

## Disclosure

All authors have reviewed the final manuscript.

## Ethics Statement

The authors have nothing to report.

## Consent

The authors have nothing to report.

## Conflicts of Interest

The authors declare no conflicts of interest.

## Data Availability

Data sharing not applicable to this article as no datasets were generated or analysed during the current study.
